# Technology Matters: Online Support and Intervention (OSI) for child anxiety problems – an example of the journey from research to practice

**DOI:** 10.1111/camh.12775

**Published:** 2025-04-03

**Authors:** Chloe Chessell, Rachel Evans, Cathy Creswell

**Affiliations:** ^1^ Department of Experimental Psychology University of Oxford Oxford UK; ^2^ Department of Psychiatry University of Oxford Oxford UK; ^3^ Koa Health London UK

**Keywords:** Online, anxiety, children, implementation, commercialisation

## Abstract

Childhood anxiety problems are prevalent and impairing, yet many children are unable to access evidence‐based treatment (i.e. cognitive behavioural therapy, CBT). Digitally augmented psychological interventions represent one way to help increase access to CBT for children with mental health problems, as these interventions can substantially reduce the amount of therapist time required to deliver the intervention, as well as bringing a range of other potential advantages for therapists and families. Online Support and Intervention (OSI) is an example of a brief digitally augmented, therapist‐supported, parent‐led CBT intervention for child anxiety problems that is now being commissioned and delivered in child mental health services. This article outlines the journey of OSI from research to implementation into routine clinical practice and highlights key considerations for translating digitally augmented mental health interventions into routine care in child mental health services.

## Background

Cognitive behavioural therapy (CBT) is an effective treatment for preadolescent children with anxiety problems (James, Reardon, Soler, James, & Creswell, 2020); however, demand often exceeds available clinical resources. Digitally augmented psychological interventions can address this gap by substantially reducing the amount of therapist time required to deliver the intervention while also ensuring fidelity of treatment delivery, providing flexibility and convenience for families, and capturing in‐built data to ensure effective outcome monitoring.

## Online Support and Intervention (OSI) for child anxiety problems

OSI is a *brief* digitally augmented, therapist‐supported, parent‐led CBT intervention that is now being commissioned in child mental health services in England. Parents complete eight online modules (each accompanied by a 20‐minute remote therapist appointment) focused on how to help their children overcome anxiety problems (Hill, Chessell, Percy, & Creswell, 2022). A large pragmatic randomised controlled trial (RCT) comparing OSI to usual (predominantly CBT) treatment in 34 UK mental health services found it to be effective and likely cost‐effective, requiring only 59% of usual treatment time to deliver, without compromising clinical outcomes or parent and therapist acceptability (Creswell et al., 2024). Following the trial, participating services were able to use OSI free‐of‐charge while we worked with a commercial partner (Koa Health) to develop arrangements for sustainable and scalable implementation going forward. Given this journey of OSI from research to practice, this article will reflect on the key considerations for translating digitally augmented mental health interventions into routine care in child mental health services. Quotes from therapists involved in the delivery of OSI in routine care (that were collected as part of a wider qualitative research project) are provided for illustrative purposes.

## Translating digitally augmented mental health interventions into routine child mental health services

### Digitally augmented interventions should be co‐designed with families and therapists

Co‐design has been central to the development of OSI and its underpinning work. The therapist‐guided, parent‐led CBT approach that OSI was based on was developed in direct response to therapists' requests for *brief* interventions that could achieve good outcomes. After establishing feasibility and effectiveness of a book‐based version (Thirlwall et al., 2013), we recognised the need to further increase accessibility and efficiency, without compromising clinical effects. This led to the development of an online platform which offers more accessible content (e.g. written and audio form, videos/animations, interactive activities) and additional benefits for users (e.g. built in routine outcome measures, ROMs). OSI was developed through extensive co‐design workshops and iterative usability testing with families and therapists (Hill, Reardon, Taylor, & Creswell, 2022). Thie use of a co‐design approach has proved invaluable; therapists using OSI frequently share positive feedback of their own, and parents', experiences that directly reflect decisions made within the codesign process: ‘it's very accessible for lots of families that we have in our service … it has certainly been streamlined, a low reading age, it's more interactive, you can listen obviously to the audio’ *[Therapist]*. Importantly, we do not consider co‐design a static process, rather a *continual* process where we, as intervention developers, continue to listen to and act on the needs of parents (e.g. via feedback collated within OSI) and therapists (e.g. via training events, email, and qualitative interviews). Specifically, parents' and therapists' feedback is used to improve the design features of OSI (rather than to alter the core treatment content) to ensure maximum usability for end users (while retaining fidelity to the evaluated treatment content).

### Understand the mental health service context and develop appropriate infrastructure to support the translation of the intervention into routine care

To effectively translate digitally augmented interventions into routine practice, it is essential to understand and respond to the mental health service contexts. In the United Kingdom, while therapists often report an innovative service culture, ‘we're quite an open service I guess to ideas and open with researchers’ *[Therapist]*, they frequently face time constraints, resource limitations and competing demands that hinder change: ‘clinicians are very busy, often working in quite stretched services and introducing a new treatment … can be sometimes met with a little bit of resistance … even though the benefit of OSI is that it reduces a lot of clinician time and is really efficient way of working’ *[Therapist]*. To address these challenges, intervention developers must provide a clear rationale for embedding new interventions in routine practice and develop appropriate supporting infrastructure. We discuss ways we have addressed these challenges in turn below.

### Strong evidence base and alignment with national clinical guidelines

Many clinical teams have been eager to incorporate OSI into their routine practice despite being over‐stretched. This enthusiasm stems from its strong evidence base, which aligns with their commitment to offering evidence‐based interventions. Additionally, the in‐built routine outcome measures (ROMs) within OSI allow services to contribute to ongoing evaluations and review their own outcomes. Recognition of OSI in a National Institute for Health and Care Excellence [NICE] Early Value Assessment [EVA], 2023, further highlights its alignment with national clinical guidelines: ‘I think cause [OSI was NICE EVA] recommended there was a push internally across the team, to kind of say … ‘are we using the digital platforms that are available?’ … and I think again moving forwards if we've got you know a combination of NICE guidance, parent feedback, clinician feedback, data that again supporting the efficacy of the model then … we have to embrace it, we have to provide that digital offer’ *[Therapist]*.

### Build and maintain key relationships

Throughout OSI's development, evaluation and implementation, we have developed strong relationships with clinical teams by involving them in research trials (e.g. Creswell et al., 2024) and supporting wider implementation. Key individuals often emerge as ‘OSI champions’ within these teams, becoming crucial drivers for change in resource‐constrained services. Champions are typically passionate about increasing access to CBT and promote OSI by discussing it in team meetings, supporting colleagues to deliver OSI and advocating for its sustained use to senior leadership: ‘those champions have been leading the way with it …. I think straight away they were very passionate about it and that really came through as it was sort of filtered down through the service’ *[Therapist]*.

### Provide regular training and support

Due to staff turnover and retention difficulties in UK mental health services, we provided free regular training and support to facilitate OSI's integration into routine practice while we developed plans for more sustainable, scalable roll‐out. This support included various options such as monthly online workshops, online training videos/resources, a detailed OSI manual, and a free online technical support desk. This was viewed by clinical teams as a key facilitator to embedding OSI in their service: ‘the practitioners have found the online training offered by the University of Oxford really helpful … we've also found the training video that's been available online to be really helpful and accessible for staff to access it at other times or to kind of do, yeah in a more manageable way’ *[Therapist]*. We have now built on these experiences and therapist feedback to develop and iterate a programme of training and support with Koa Health (that includes more sustainable models of training such as a ‘train the trainer’ model where cohorts of therapists are trained to deliver OSI and then supported to become OSI trainers and supervisors for incoming therapists in their service) which is being very positively received by therapists.

### Consider sustainability from the onset

Sustaining interventions must be considered from the very start of the development phase, from both developers' and services' perspectives. Academic developers can choose ‘in‐house’ (i.e. take on the role of dissemination themselves), ‘commercial’ (i.e. work with a company to facilitate dissemination) or combined approaches for large‐scale dissemination (Hill et al., 2018). For OSI, we partnered with a company to capitalise on their expertise (e.g. in marketing, contracts, maintenance of interventions) and established healthcare connections (Hill et al., 2018). From a service perspective, there is an inevitable cost given that digitally augmented interventions require hosting, maintenance and updates to continue to meet end‐user needs (Hill et al., 2018). Despite efforts to minimise expenses, these costs can hinder translation into practice as the potential pay‐offs from their use will take time to come into effect. Currently, there is a lack of ring‐fenced funding for digital mental health interventions in child mental health services, highlighting a need for future investment. For OSI, commissioning and provision have been facilitated for some services through participation in research trials (e.g. Creswell et al., 2024) and free interim use, allowing them to integrate the intervention and build a business case for continued use.

## Conclusion

Digitally augmented interventions are a potential key way to increase accessibility to effective psychological therapies for children and families who could benefit from them. To translate these interventions into routine mental health services, it is critical that intervention developers co‐design (and continue to maximise the usability of) digitally augmented interventions, understand the challenges of the context in which their intervention will be based, and develop appropriate infrastructure to support and sustain the use of the intervention in routine services. Moving forward, given the increasing global demand for accessible effective mental health provision, it will be useful for those implementing digitally augmented interventions to build on the learning from implementation efforts in the United Kingdom, and to establish the extent to which this learning applies in diverse settings, to maximise the potential to substantially increase access to mental health provision on a global scale.

## Funding information

The work described in this paper has been supported by the National Institute for Health and Care Research (NIHR) Applied Research Collaboration Oxford and Thames Valley at Oxford Health NHS Foundation Trust, the Oxford Health NIHR Biomedical Research Council and the Paul Foundation. The views expressed are those of the authors and not necessarily those of the NHS, the NIHR or the Department of Health and Social Care.

## Ethics statement

The illustrative quotes outlined in this article were collected as part of a wider qualitative research project, which received ethical approval from the University of Oxford Medical Sciences Interdivisional Research Ethics Committee (Ethics Approval Reference: R85018/RE001, 08/03/24). Informed consent was obtained from therapists for illustrative quotes to be used in research outputs.

## Conflict of interest statement

CCr is one of the developers of OSI; however, the author does not receive any personal financial gain from the sales of OSI. The remaining authors have declared that they have no competing or potential conflicts of interest.

## Data Availability

Data sharing is not applicable to this article as no new data were created or analysed.
